# Melatonin Treatment at Dry-off Reduces Postpartum Shedding of Coccidia in Primiparous Dairy Cows and Their Calves

**DOI:** 10.3390/ani14233534

**Published:** 2024-12-06

**Authors:** Fernando López-Gatius, Sergi Ganau, María Mora-García, Irina Garcia-Ispierto

**Affiliations:** 1 Agrotecnio Centre, 25198 Lleida, Spain; irina.garcia@udl.cat; 2 Transfer in Bovine Reproduction SLu, 22300 Barbastro, Spain; 3 Granja Sant Josep, La Melusa, 22549 Tamarite, Spain; 4 Department of Animal Science, University of Lleida, 25198 Lleida, Spain; mariaolle15@gmail.com

**Keywords:** cattle diseases, circadian rhythm, colostrum quality, eimeriosis, intestinal pathogens, vaccination program

## Abstract

Coccidiosis is a protozoan disease that causes diarrhea in cattle. This study examines the impact of treating pregnant cows at dry-off with melatonin on postpartum coccidia excretion in dams and their calves. The study population comprised 106 primiparous lactating dairy cows: 53 controls and 53 receiving melatonin on days 220–226 of gestation, plus 99 calves: 52 born from control and 47 from treated dams. Feces samples were collected from each dam on gestation days 220–226 and on days 10–16 and 30–36 postpartum; and from each calf on days 10–16 and 30–36 of age. Postpartum rates of high excretion of oocysts per gram (OPG) (feces counts > 5000) were significantly lower (*p* < 0.01) in treated than control dams. Low excretion rates of OPG (<2000) were significantly higher (*p* < 0.01) in the melatonin treatment than control groups in dams on days 30–36 of lactation and in calves at 10–16 and 30–36 days of life. In conclusion, melatonin treatment in lactating cows at dry-off reduced coccidia shedding in dams and their calves during the early postpartum period.

## Simple Summary

Coccidiosis is a protozoan parasite disease that causes diarrhea in livestock worldwide. Because of their underdeveloped immune system, young bovine calves are highly susceptible. In calves of up to two months of age, diarrhea can lead to death if not managed promptly. Melatonin, synthesized from the essential amino acid tryptophan in the pineal gland, shows immunomodulatory properties. In fact, exogenous melatonin has been proposed as therapy to trigger an immune response against infectious diseases. Immunosuppression around the time of delivery has been extensively associated with early postpartum diseases of infectious cause in cattle. As public concerns about the use of antibiotics limit preventive actions, the objective of the present study was to explore the effects of melatonin treatment two months before expected parturition as a way of boosting the immune system of the dams and their calves. Treatment significantly reduced coccidia shedding in both dams and their calves during the early postpartum period.

## 1. Introduction

Coccidiosis (aka eimeriosis) is a protozoan disease caused by several *Eimeria* species (Apicomplexa: Eimeriidae) that produces diarrhea in livestock worldwide. Several species of *Eimeria* are considered pathogenic in cattle, mostly *E. bovis* and *E. zuernii* [1,2,3]. Coccidiosis has profound impacts on the health and productivity of cattle and leads to economic losses as the consequence of diminished growth rates, weight loss, reduced milk production, and increased mortality [1,2,3]. High-intensity dairy farming practices exacerbate the risk of coccidiosis due to factors such as overcrowding and stress, which facilitate the spread of *Eimeria* oocysts [4,5,6]. Young bovine calves are highly susceptible due to their underdeveloped immune system [3,6]. Significant correlation between the presence of pathogenic *Eimeria* species and diarrhea has been observed in calves of up to two months of age [7], possibly leading to death if not managed promptly [8]. However, no anticoccidial drug is completely effective at avoiding adverse effects on calf health and productivity [3]. In addition, public concerns regarding the use of antibiotics have meant that preventive actions such as prophylactic or metaphylactic treatments are usually avoided. While the old saying “prevention is better than cure” endorses the improvement of the health of mankind through vaccination to control infectious diseases [9], this may also be appropriate today for diseases of protozoan origin in cattle. Notwithstanding, given the current lack of a vaccine for bovine coccidiosis, any strategy to minimize infection pressure is welcome.

Melatonin (N-acetyl-5-methoxytryptamine), first isolated in 1958 from the bovine pineal gland [10], is synthesized from the essential amino acid tryptophan [11,12]. Although mainly synthesized in this gland, melatonin has also been found in numerous non-endocrine sites [13,14,15], including the immune system [16,17,18], and is also widely present in phylogenetically distant organisms, from plants, bacteria, and fungi to vertebrates [19,20,21]. The ubiquitous distribution of melatonin in nature reflects its broad functional versatility; for example, its antioxidant and immunomodulatory properties, among others [15,22,23]. However, the most important role of pineal melatonin is the regulation of seasonal physiological functions [23,24,25]. Seasonal-breeding species use the photoperiod, the predictable annual cycle of a favorable length of day, as a calendar [26]. As melatonin production by the pineal gland is proportional to night length and light exposure suppresses its synthesis, in mammals this molecule acts as a biological signal of darkness [27,28,29]. In fact, changes in day-length drive reproductive rhythms in seasonal breeding animals that live outside tropical zones [30,31,32]. Consequently, exogenous melatonin has been extensively used to control reproduction in small ruminants [33,34,35] and buffaloes outside the breeding season [36,37,38]. While cows are not seasonal breeders, the light/dark ratio may still influence endocrine patterns of gestation [39], and a negative photoperiod (decreasing day-length) correlates with the incidence of twin pregnancies [40,41,42]. Despite this, the potential benefits of exogenous melatonin have been scarcely addressed in cattle. In a previous study, we found that melatonin treatment at dry-off improved the postpartum reproductive performance of high-producing dairy cows under heat stress conditions [43]. Treatment was administered during the longest days of the year (long photoperiod, May–August), favoring the effects of exogenous melatonin in animals that were melatonin-deficient [43].

Peripartum immunosuppression has been extensively associated with early postpartum infectious diseases in cattle [44,45,46]. Recently, due to its antioxidant and immunomodulatory effects, exogenous melatonin has been proposed as therapy to boost the immune response against viral, bacterial, and parasite infections [47,48,49]. In effect, exogenous melatonin emerges as a new strategy for livestock production [50]. For example, melatonin administered to pregnant ewes was noted to reduce oocyst excretion after experimental infection with *Eimeria* species [51]. The objective of the present study was to examine the impact of melatonin treatment at dry-off on postpartum *Eimeria* oocyst excretion in primiparous dairy cows and their calves.

## 2. Materials and Methods

### 2.1. Cows and Herd Management

This prospective observational cohort study was conducted in a Holstein high-producing dairy herd in north-eastern Spain (latitude 41.13 N, longitude −2.4 E). During the period of melatonin treatment from 17 July 2023 to 11 March 2024, the mean number of lactating cows in the herd was 4358, the mean culling rate was 23.5%, and daily milk production was 46.2 kg per milking cow. The cows were milked thrice daily and fed complete rations, which were the same from day 30 postpartum until dry-off. Lactating cows were grouped according to number of pregnancies into primiparous, secundiparous, or in their third lactation or more. Dry cows were kept separately and marked as the “parturition group” 7–25 days before the expected date of calving, according to whether or not they were carrying twins. The herd was maintained on a weekly reproductive health program. This involved pregnancy diagnosis 28–34 days post-insemination when the number of embryos was recorded.

Only healthy cows were included in the study, as indicated by a body condition score of 2–3.5 on a scale of 1 to 5 [52] and lack of clinical signs of disease at the time of treatment. Exclusion criteria were mastitis, lameness, and digestive disorders. All animals were reared within the herd.

#### 2.1.1. Immunizations

The vaccination program for the herd is shown in Table 1. In pregnant cows and heifers, all vaccines were administered from days 227 to 241 of gestation.

#### 2.1.2. Management of Colostrum Feeding and of Diarrhea Signs

Low bacterial count, non-mastitic, high-quality colostrum pooled from five cows in their third or more lactation was pasteurized at 65 °C for 60 min and kept at 5 °C for no more than four days in labeled and dated four-liter containers. Immunoglobulin (Ig) colostrum contents were estimated using a Brix refractometer (ATAGO Brix refractometer PAL-1; Saitama, Japan). Only colostrum with a value greater than 22% Brix, equivalent to >50 mg/mL of Ig [53,54], was stored to be fed to the calves. Newborn calves were given four liters of warmed colostrum (39 °C) via an esophageal tube during the first six hours of life, and colostrum feedings are continued until they received six liters on the first day. Calf total serum protein was measured using the Brix refractometer within the second day of birth. A value greater than 8.5% Brix indicates good transfer of passive immunity in calves [55,56].

Two feedings of four liters each of warmed (39 °C) non-mastitic fresh milk (pooled from five pluriparous cows taken during the second through sixth milking postpartum), pasteurized at 60 °C for 60 min and stored in the fridge, were fed to the calves for fourteen days following colostrum consumption. Calves were then fed a milk substitute with water and solid food available.

Calves with low-consistency feces received an isotonic balanced electrolyte solution. If feces became watery with the presence or not of blood and/or intestinal tissue, pectin was added to the electrolyte solution. Antibiotic treatment was added to the fluid therapy for diarrheic calves with pyrexia (>39.5 °C). Fecal samples from all calves experiencing watery diarrhea were collected to quantify the presence of *Eimeria* oocysts in a commercial laboratory. In these tests, all samples showed coccidia oocysts during the year prior to the study. In addition, diarrhea and non-diarrhea fecal samples from six calves were pooled every two weeks to detect the possible presence of *Cryptosporidium* species. The overall prevalence of these species was 0% during the study period.

### 2.2. Experimental Design

Our experiment was adapted to the routine practices of the herd. Parturitions of the study population were scheduled for the cool period of the year. We had two reasons for this. First of all, in our geographical region there are only two clearly differentiated weather periods, warm (May–September) and cool (October–April). During the cool period, reproductive variables improve, and interactions among postpartum reproductive disorders are greatly reduced [57,58,59]. And second, during the parturition period, associated with the winter solstice, i.e., during the shortest days of the year, endogenous melatonin production is at its highest [27,28,29]. Thus, reduced light conditions at treatment will be a harder test to detect the possible beneficial effects of exogenous melatonin, as endogenous melatonin levels in the animals will already be high. Under these conditions, the impacts of exogenous melatonin on maternal pathophysiology, if any, should be unveiled.

To homogenize the study population and avoid the possible effects of calf sex on the level of coccidia excretion, only primiparous cows that became pregnant using sexed semen carrying singletons were included in the study. The presence of singletons was detected by ultrasound on days 28–34 of gestation and confirmed three weeks later. Pregnancy was again confirmed before melatonin administration. Cows were randomly assigned to a control (*n* = 55) or melatonin (*n* = 55) group. Cows in the latter group were given 12 slow-releasing subcutaneous implants of the hormone (Melovine^®^, CEVA Salud Animal, Barcelona, Spain) on days 220–226 of gestation as described previously [43]. In this previous study, we estimated the half-life of exogenous melatonin as 116 days. Thus, its expected effect was beyond the parturition period. Each implant contained 18 mg of melatonin and was administered using an implanter supplied by the pharmaceutical company to an area of free-moving skin between the thighs, dorsal to the base of the udder [43].

Four cows were removed from the herd early postpartum due to low milk production, two controls and two melatonin-treated cows. Thus, the final study population was made up of 53 control and 53 melatonin-treated cows. From each dam, feces samples were collected at the time points: gestation days 220–226 and days 10–16 and 30–36 postpartum. In calves, samples were collected on days 10–16 and 30–36 of age. Feces were collected directly from the rectum (at least 20 g) into sterile screw-capped plastic disposable containers. Diarrhea, best judged by feeling feces [60], was classified upon collection into three categories: no diarrhea with a water content around 70–80% (firm pulp); mild diarrhea with a water content around 80–90% (lacking consistency); and severe diarrhea with a water content over 90% (watery feces) with or without the presence of blood and/or intestinal tissue.

#### Coprological Test

Fecal samples were stored at 5 °C and analyzed within a day after collection according to a modified procedure of the paraTest^®^ Vet (Ritter GmbH, Schwabmünchen, Germany). This test uses a qualitative flotation diagnostics system to detect coccidia oocysts. The flotation medium (31% sodium nitrate solution) has a specific weight of 1.2, and the test is based on test tube and cover slip procedures. Briefly, 3 g of feces from each sample is mixed with 42 mL of flotation fluid. The suspension is left undisturbed, allowing a convex meniscus to form at the top of the test tube. A coverslip is placed to float on the meniscus for 15 min. The coverslip is then carefully removed and placed on a slide for microscopy examination. By means of flotation, the parasite oocysts, if present, should remain on the cover slip. However, as noted above, the prevalence of coccidia is high in the herd. In addition, to assess the possibility of reduced oocyst excretion after treatment, the level of this excretion was quantified. For this purpose, the use of the cover slip was eliminated from the qualitative test so that we could count the oocysts obtained from the supernatant fluid at the meniscus level in a McMaster counting chamber. In this procedure, 0.3 mL of fluid was examined within the McMaster chamber (with two counting areas) under the microscope at 100 × magnification. Based on this, the detection limit of oocysts per gram (OPG) of feces was calculated as (45/3)/0.3 = 50 OPG, whereas total OPG were calculated as (n/2 counting areas) × dilution (1/15) × volume in a counting area (0.15) × 300.

According to OPG values, fecal samples were classified into three categories: 1: low excretion (<2000 OPG); 2: medium excretion (between 2000 and 5000 OPG); and 3: high excretion (>5000 OPG).

### 2.3. Data Collection and Statistical Analysis

All data are expressed as the mean ± standard deviation (SD) and range (in parentheses). The data obtained from each dam were: treatment group (control versus melatonin), parturition date, dystocia, stillbirth, calf sex, placenta retention (fetal membranes retained longer than 12 h after parturition), hypocalcemia, ketosis (diagnosed during the first or second week postpartum), metritis (diagnosed during the first or second week postpartum in cows not suffering placental retention), endometritis (diagnosed during the third week postpartum), milk production on day 50 postpartum (mean production in the seven days before), and diarrhea (absence, mild, or severe) and concentrations of excreted *Eimeria* oocysts for each time point. In each calf, the data recorded were treatment group (control versus melatonin), weight on days 20–26 of life, and diarrhea (absence, mild, or severe) and concentrations of excreted *Eimeria* oocysts for each time-point sample. When appropriate, possible differences were analyzed by the student’s *t*-test (means) or by the chi-square test (proportions). Significance was set at *p* < 0.05.

## 3. Results

Mean milk production on day 50 postpartum for all cows was 47.9 ± 5.3 (34.3–61.2) kg: 48 ± 5.4 (34.9–61.2) kg for the control and 47.8 ± 5.2 (34.3–56.6) kg for the melatonin group. Mean weight on days 20–26 of life for all calves was 55.5 ± 5.8 (42–69) kg: 55.8 ± 5.8 (45.5–69) kg for the control group and 55.2 ± 5.9 (42–66) kg for the melatonin group. Through the student’s *t*-test, no significant differences in these variables were detected between the control and melatonin groups.

All 53 control and 53 melatonin-treated cows remained in the herd throughout the study period. Dystocia, hypocalcemia, and endometritis were not recorded in any cow. Five cows (one in control and four in melatonin) delivered male calves, and two further cows (in melatonin) had a stillborn calf at parturition. Data from these seven cows were kept for analysis, whereas data from their calves were removed from the study. The remaining 99 calves (52 in control and 47 in melatonin) were females and remained alive at 120 days of age. Five cows (two in control and three in melatonin) underwent placenta retention; two control cows had metritis, and ketosis was diagnosed in four further cows (three in control and one in melatonin). These disorders could not be associated with treatment or with postpartum oocyst counts.

**Figure 1:**
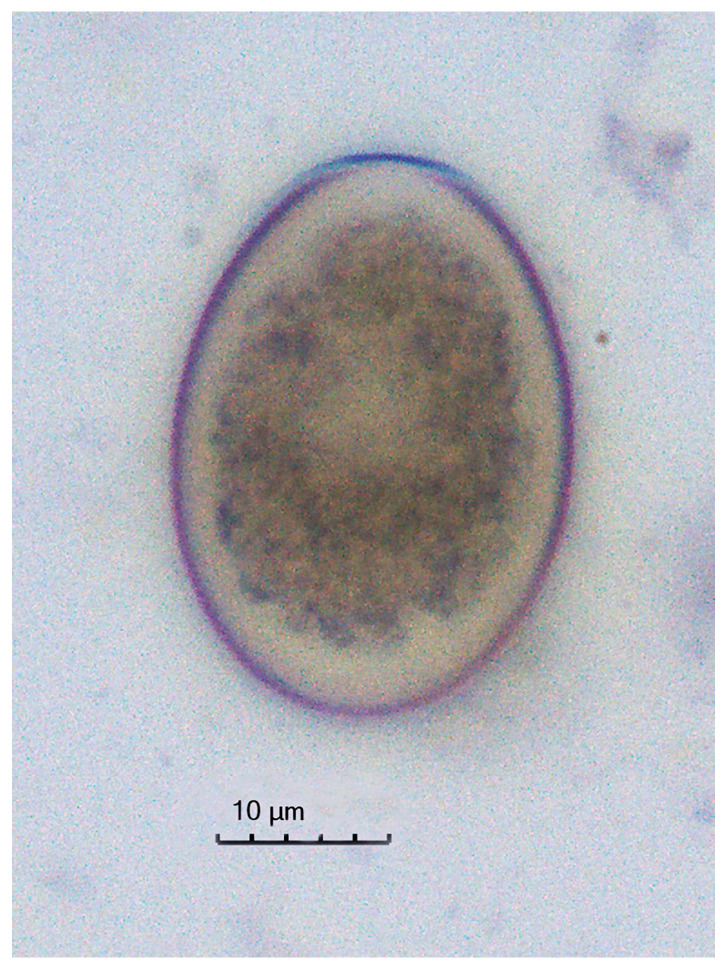
Unsporulated *Eimeria bovis*-like oocyst at 400 × magnification from a sample obtained from a dam at 10 days postpartum.

Although identification of bovine *Eimeria* species was not the objective of the present study, *Eimeria bovis*-like oocysts were predominant in all samples. The oocysts were ovoid forms, yellowish to pale brown, and featuring a smooth bilayer wall with a micropyle at the narrow end (Figure 1) [61,62].

Diarrhea signs were not observed in any dam, whereas pyrexia was not recorded in any calf experiencing severe diarrhea. On days 10–16 of life, mild diarrhea was recorded in 13 (13.1%) calves (8 in control and 5 in melatonin), and severe diarrhea in 10 (10.1%) calves (4 in control and 6 in melatonin), whereas on days 30–36 of life, mild diarrhea was noted in 9 (9.1%) calves (4 in control and 5 in melatonin), and severe diarrhea in 2 (2%) calves (2 in control and 0 in melatonin).

Mean oocyst counts on days 220–226 of gestation for all cows were 2959 ± 1840 (50–6100) oocysts: 2980 ± 1824 (50–6100) oocysts for the control and 2936 ± 1876 (50–6100) oocysts for the melatonin group. Table 2 summarizes the effects of melatonin treatment on the categories of oocyst excretion, along with diarrhea signs for each time point in dams and calves. Given the low incidence of diarrhea in both the control and melatonin groups, cases of diarrhea were grouped for each time point. Postpartum rates of high excretion of oocysts were significantly (*p* < 0.01) lower for the melatonin-treated than control dams. The rates of low oocyst excretion were significantly (*p* < 0.01) higher for the melatonin than control group dams at days 30–36 of lactation and in calves at 10–16 and 30–36 days of life.

## 4. Discussion

The results of the present study offer the first supporting evidence that melatonin treatment at dry-off may reduce the postpartum shedding of coccidia in dams and their calves. It should be noted that these results were obtained by administering treatment during the period of maximum endogenous melatonin production, that is, the shortest days of the year (short photoperiod). While proper colostrum management and vaccination programs reduced the risk of infectious enteropathogenesis in calves, excretion of *Eimeria* oocysts was ubiquitous in this herd. In effect, coccidia were observed in all samples, though values showed high variation. The practice of vaccinating pregnant cows during the weeks preceding parturition has been associated with increased concentrations of antigen-specific protective colostrum antibodies [63,64,65]. In addition, older cows longer exposed to farm-specific pathogens produce higher-quality colostrum [66,67,68]. Presumably, the good colostrum quality in this herd prevented co-infection of *Eimeria* species with other agents. In this context, reduced coccidia excretion in the dams and their calves must have been the outcome of melatonin treatment. These results indicate that exogenous melatonin improved the immunocompetence of both dams and their fetuses during late pregnancy, which is when the immune response of the dam improves as the fetal immune system matures [69,70]. However, this study has a limitation that should be considered. Determination of anti-*Eimeria* defenses in newborn calves should reinforce results from calves. More work is needed to determine the exact mechanism whereby melatonin enhances the immune response of an advanced pregnant cow and its fetus against protozoan infections.

Although no oocyst differentiation, including the proportion of pathogenic *Eimeria* species, was performed, high oocyst counts were associated with the incidence of diarrhea, consistent with previous studies [6,7,8]. In fact, the categorization of the number of oocysts proved to be useful in confirming the positive effects of treatment by reducing coccidial excretion. Endogenous melatonin production varies throughout the year. Hence, we would expect that melatonin treatment around the summer solstice, when days are longest (long photoperiod) and endogenous melatonin is minimal, will result in an enhanced immune response in a cow and her calf. It would also be interesting to see if exogenous melatonin can also reduce the risk of cryptosporidiosis, another common protozoan parasite disease frequently associated with neonatal diarrhea [71,72,73]. Of course, calf diarrhea has a multifactorial etiology [71,72], so the effect of melatonin on newborn health would need to be investigated.

As expected, melatonin treatment at dry-off had no influence on subsequent milk production, in agreement with prior observations [43]. Exogenous melatonin reduces plasma prolactin concentrations [43,74], and this may lead to diminished milk production [74]. Our results reinforce the notion that the dry-off period is the optimal time to treat lactating animals, as it is at this time point that this hormone treatment does not affect milk production, and peripartum immunosuppression in the cow can be overcome. However, under certain circumstances, such as high somatic cell counts or mastitis, exogenous melatonin may reduce somatic cell counts and improve milk quality by increasing its lactose and protein contents [75,76,77].

## 5. Conclusions

In a dairy herd ubiquitously excreting coccidia and showing a low risk of co-infection, the treatment of lactating cows with melatonin at dry-off was found here to reduce coccidia shedding in dams and their calves during the early postpartum period. This treatment was administered during the peak period of endogenous melatonin production, that is, during the shortest days of the year. We would in effect expect an improved response to treatment during the longest days of the year, when endogenous melatonin production is at its minimum.

## Figures and Tables

**Table 1:** Vaccinations.

Disease (Agent)	Vaccine (Type/Route)	Age of Initial Vaccination/Booster
Parainfluenza-3 virus (PI-3V) strain RLB103	Modified live Intranasal	Calves: 5 to 11 days of age
Respiratory syncytial virus (BRSV) Strain 375	Modified live Intranasal	Calves: 5 to 11 days of age
Parainfluenza-3 virus (PI-3V) strain BIO-23	Killed Subcutaneous	Calves: 12 to 18 days of age. Revaccination at 40 to 46 days of age, and at 300 to 307 days of age. Pregnant cows and heifers: Days 241–247 of gestation. Revaccination every gestation.
Respiratory syncytial virus (BRSV) Strain 375	Killed Subcutaneous	Calves: 12 to 18 days of age. Revaccination at 40 to 46 days of age. Pregnant cows and heifers: Days 241–247 of gestation. Revaccination every gestation.
Pasteurellosis *P. multocida* serotypes A and 6B	Killed Subcutaneous	Calves: 12 to 18 days of age. Revaccination at 40 to 46 days of age.
Infectious bovine rhinotracheitis (IBR) Bovine herpesvirus type 1 Strain GK/D	Modified live Intranasal	Heifers: 120 to 126 days of age.
Clostridiosis *C. perfringens, C. Chauvoei, C. septicum, C. tetani, C. haemoliticum*	Toxoids Subcutaneous	Heifers: 140 to 147 days of age. Revaccination at 168 to 174 days of age, and at 300 to 306 days of age. Pregnant cows and heifers: Days 241–247 of gestation. Revaccination every gestation.
Infectious bovine rhinotracheitis (IBR) Bovine herpesvirus type 1 strain GK/D	Killed Intramuscular	Heifers: 300 to 306 days of age. Pregnant cows and heifers: Days 234–240 of gestation. Revaccination every gestation.
Bovine viral diarrhea (BVD) Virus 1 strain KE-9 Virus 2 strain NY-93	Modified live Intramuscular	Heifers: 300 to 306 days of age. Pregnant cows and heifers: Days 234–240 of gestation. Revaccination every gestation.
Bovine viral diarrhea (BVD) type 1 Strain C-86	Killed Intramuscular	Pregnant cows and heifers: Days 234–240 of gestation. Revaccination every gestation.
Neonatal diarrhea *E. coli* Bovine rotavirus Bovine coronavirus	Killed Intramuscular	Pregnant cows and heifers: Days 227–233 of gestation. Revaccination every gestation.

**Table 2:** Effects of melatonin treatment on *Eimeria* oocyst excretion and diarrhea signs in dams (*n* = 106) and their calves (*n* = 99).

Sampling Time	Oocyst Excretion ^(a)^	Controls	Melatonin	No Diarrhea ^(b)^	Mild Diarrhea	Severe Diarrhea
Dams						
Gestation day 220–226	low medium high	16/53 (30.2%) 24/53 (45.3%) 13/53 (24.5%)	19/53 (35.8%) 25/53 (47.2%) 9/53 (17.0%)	35/35 (100%) 49/49 (100%) 22/22 (100%)	- - -	- - -
Postpartum day 10–16	low medium high	18/53 (34.0%) 25/53 (47.2%) 10/53 (18.9%) *	26/53 (49.1%) 26/53 (49.1%) 1/53 (1.9%) **	36/36 (100%) 50/50 (100%) 11/11 (100%)	- - -	- - -
Postpartum day 30–36	low medium high	18/53 (34.0%) * 23/53 (43.4%) 12/53 (22.6%) *	32/53 (60.3%) ** 20/53 (37.7%) 1/53 (1.9%) **	50/50 (100%) 43/43 (100%) 13/13 (100%)	- - -	- - -
Calves						
Time of life 10–16 days	low medium high	6/52 (11.5%) * 30/52 (57.7%) 16/52 (30.8%)	16/47 (34%) ** 20/47 (42.6%) 11/47 (23.4%)	22/22 (100%) 41/50 (82%) 13/27 (48.2%)	0/22 (0%) 9/50 (18%) 4/27 (14.8%)	0/22 (0%) 0/50 (0%) 10/27 (37%)
Time of life 30–36 days	low medium high	9/52 (17.3%) * 29/52 (64.4%) 7/52 (15.6%)	20/47 (43.5%) ** 24/47 (52.2%) 2/47 (4.3%)	29/29 (100%) 46/53 (86.8%) 5/9 (55.6%)	0/29 (0%) 7/53 (13.2%) 2/9 (22.2%)	0/29 (0%) 0/53 (0%) 2/9 (22.2%)

^(a)^ Values with different superscripts within rows denote significant differences detected by the chi-square test (*, **: *p* < 0.01). ^(b)^ Diarrhea was classified upon sample collection into three categories according to how feces felt as: no diarrhea (firm pulp), mild diarrhea (lacking consistency), and severe diarrhea (watery feces). As there were no significant differences between control and treated animals, cases of diarrhea were grouped for each time point.

## Data Availability

The data are not publicly available due to privacy and 3rd party agreements.
